# Hypertension and Pathogenic hAPP Independently Induce White Matter Astrocytosis and Cognitive Impairment in the Rat

**DOI:** 10.3389/fnagi.2020.00082

**Published:** 2020-04-15

**Authors:** Alexander Levit, Sonny Cheng, Olivia Hough, Qingfan Liu, Yuksel Agca, Cansu Agca, Vladimir Hachinski, Shawn N. Whitehead

**Affiliations:** ^1^Vulnerable Brain Lab, Department of Anatomy and Cell Biology, Schulich School of Medicine & Dentistry, Western University, London, ON, Canada; ^2^Department of Veterinary Pathobiology, College of Veterinary Medicine, University of Missouri, Columbia, MO, United States; ^3^Department of Clinical Neurological Sciences, University Hospital, Western University, London, ON, Canada

**Keywords:** hypertension, amyloid, astrocytes, microglia, white matter, cognitive function, transgenic rat

## Abstract

Hypertension is recognized as a risk factor for Alzheimer disease, but the causal link remains undetermined. Although astrocytes and microglia play an important role in maintaining the neurovascular unit, astrocytes and microglia have been understudied in comorbid models of hypertension and Alzheimer disease. In this study, male transgenic Fischer 344 rats (TgAPP21) overexpressing a pathogenic human amyloid precursor protein received 8 weeks of Angiotensin II infusion to increase blood pressure, and the rats were evaluated for astrocytosis, microgliosis, and cognitive function. A linear relationship between astrocytosis and blood pressure was observed in the corpus callosum and cingulum of wildtype rats, with hypertensive wildtype rats matching the elevated baseline astrocytosis seen in normotensive transgenic rats. In contrast, hypertensive transgenic rats did not demonstrate a further increase of astrocytosis, suggesting a deficient response. Angiotensin II infusion did not affect activation of microglia, which were elevated in the white matter and hippocampus of transgenic rats. Angiotensin II infusion did impair both wildtype and transgenic rats’ executive functions in the Morris Water Maze. These results present important implications for the interaction between hypertension and pathogenic human amyloid precursor protein expression, as Angiotensin II infusion produced cognitive impairments in both genotypes, but transgenic rats were additionally impaired in developing a normal astrocytic response to elevated blood pressure.

## Introduction

Cerebral vascular pathology is commonly observed in autopsy-confirmed cases of Alzheimer’s disease (AD) at significantly greater rates than age-matched control cases (Toledo et al., 2013). The relationship between AD and vascular pathology has given rise to the neurovascular hypothesis of AD (Zlokovic, 2005, 2011; Kisler et al., 2017), which proposes that cerebrovascular dysregulation, including the effect of systemic hypertension on the brain, disrupts amyloid and tau protein homeostasis, leading to neuronal injury and cognitive impairment. At the same time, amyloid- and tau-mediated injury can disrupt neurovascular coupling. Central to this bi-directional hypothesis is the neurovascular unit, maintained in part by astrocytes and microglia. Both of these glial cell types demonstrate important physiological responses to both hypertension and amyloid (Prokop et al., 2013; Dunn and Nelson, 2014; Shen et al., 2015; von Bernhardi et al., 2015; Frost and Li, 2017).

Hypertension is recognized as a leading vascular risk factor for AD (Oveisgharan and Hachinski, 2010; Iadecola, 2014; Livingston et al., 2017) but studies on whether anti-hypertensive therapy can offer cognitive protection have had mixed results (Lithell et al., 2004; Peters et al., 2008; McGuinness et al., 2009; Perrotta et al., 2016). A recent meta-analysis of prospective cohort studies showed a reduced risk of dementia with use of any antihypertensive medication (Ding et al., 2019), suggesting a benefit that was independent of drug class. The recent SPRINT-MIND trial demonstrated a reduced incidence of cognitive impairment in the intensive blood pressure control group when compared to standard treatment group (Williamson et al., 2019), further supporting a direct relationship between neurodegenerative disease and elevated blood pressure.

The importance of hypertension as a risk factor for AD is further supported by the strong link between hypertension and abnormal white matter changes presenting as leukoaraiosis, also known as white matter hyperintensities (van Dijk et al., 2004). Hypertension is the leading risk factor for leukoaraiosis, and in turn, leukoaraiosis is an important predictor for dementias, including AD (Debette and Markus, 2010). These white matter disruptions are associated with disruptions of white matter integrity (O’Sullivan et al., 2004; Altamura et al., 2016) and can cause impairments of executive functions such as working memory and behavioral flexibility (O’Sullivan et al., 2004). These changes are also observed in patients with hypertension (Raz et al., 2003; Vicario et al., 2005; Li et al., 2016). Hypertension disrupts astrocytic polarity (Yamagata et al., 1997; Vitaioli et al., 2004; Tomassoni et al., 2010; Wang et al., 2018), which may be an initiating factor in the development of leukoaraiosis (Huang et al., 2018). Thus, an experimental model that captures the effects of hypertension on white matter astrocytes will be crucial to investigating possible causal relationships between hypertension and AD.

Previous animal models of comorbid hypertension and AD have demonstrated that hypertension does exacerbate amyloidopathies and cognitive impairment (Díaz-Ruiz et al., 2009; Gentile et al., 2009; Carnevale et al., 2012, 2016; Csiszar et al., 2013; Cifuentes et al., 2015), but these studies did not present data on executive function and white matter gliosis. In the present study, we investigated the impact of hypertension on the transgenic Fischer 344 rat (TgAPP21) which overexpresses a pathogenic variant of the human amyloid precursor protein (hAPP; swe/ind mutations) (Agca et al., 2008), focusing on astrocytes, microglia, and executive function. Cerebral amyloid pathology does not occur spontaneously in TgAPP21 but can be induced (Agca et al., 2008; Rosen et al., 2012; Silverberg et al., 2015). Furthermore, TgAPP21 demonstrate a greater vulnerability to cerebrovascular injury than age-matched wildtype controls (Levit et al., 2017). Thus, TgAPP21 are ideal for modeling the roles of hypertension and glial cells in the early pre-plaque stages of AD. For 8 weeks, 8–10 month old male wildtype and transgenic rats were infused with either normal saline (Wt and Tg rats) or Angiotensin II (AngII; Wt-AngII and Tg-AngII rats) to elevate blood pressure and model the effects of hypertension (Crowley et al., 2006; Osborn et al., 2011; Lohmeier, 2012). We expected increased astrocytosis particularly in the white matter regions of Wt-AngII and Tg-AngII, accompanied by executive dysfunction, as both white matter and executive function are particularly vulnerable to hypertension (Raz et al., 2003; Vicario et al., 2005; Li et al., 2016). As both hypertension and high levels of amyloid activate both astrocytes and microglia (Vitaioli et al., 2004; Tomassoni et al., 2010; Prokop et al., 2013; Dunn and Nelson, 2014; Shen et al., 2015; von Bernhardi et al., 2015; Frost and Li, 2017; Wang et al., 2018), we expected the greatest amount of glial activity in the comorbid Tg-AngII rats.

Indeed, after 8 weeks of elevated blood pressure, we found greater astrocyte reactivity in the corpus callosum and cingulum of Wt-AngII rats than in Wt rats. The level of white matter astrocytosis in Wt-AngII rats was similar to Tg rats, which appeared to have an elevated baseline level of reactive astrocytes. However, Tg-AngII rats did not demonstrate a further increase of astrocytosis. AngII infusion did impair both Wt-AngII and Tg-AngII rats in the Morris Water Maze (MWM) adaptation of a delayed match-sample test, a spatial task that also tests working memory and behavioral flexibility. These results present important implications for the interactive effects of hypertension and genetic risk factors for AD, as AngII infusion produced cognitive impairments in both genotypes, but Tg-AngII were additionally impaired in developing a normal astrocytic response to elevated blood pressure.

## Materials and Methods

### Animals

Animal ethics and procedures were approved by the Animal Care Committee at Western University (protocol 2014-016) and are in compliance with Canadian and National Institute of Health Guides for the Care and Use of Laboratory Animals (NIH Publication #80-23). Homozygous TgAPP21 rats were studied to model the effect of increased brain concentrations of pathogenic hAPP (Agca et al., 2008). Twenty-six male wildtype Fischer 344 rats and 29 male TgAPP21 rats were aged to 7.25 months (SD = 0.55 months), weighing an average of 367 g (SD = 38 g), before osmotic pumps were implanted to deliver saline or AngII for 8 weeks. Behavioral testing was performed during the last 2 weeks of saline or AngII infusion.

### Blood Pressure Elevation and Measurement

With random allocation, 13 wildtype and 14 TgAPP21 rats were infused with normal saline (Wt, Tg); 13 Wt and 15 TgAPP21 rats were infused with AngII to elevate blood pressure (Wt-AngII, Tg-AngII) (Crowley et al., 2006; Osborn et al., 2011; Lohmeier, 2012). Osmotic pumps (Alzet, model 2004; Cupertino, CA, United States) were filled with a saline-angiotensin II solution (Sigma Aldrich, A9525; Oakville, ON, Canada) or with normal saline. The angiotensin II solutions were diluted according to lot-specific osmotic pump flow rates and individual rat weight to deliver 10,000 ng/kg/h. The pumps were implanted subcutaneously on the medial dorsum at the level of the scapulae. The pump reservoir allowed for drug or saline delivery for only 4 weeks, so pumps were replaced once to allow delivery for a total of 8 weeks. Volume pressure reading tail cuffs were used to measure arterial tail blood pressure (Kent Scientific, CODA High Throughput) (Kurtz et al., 2005; Feng et al., 2008). In between pump implantation and behavioral tests, blood pressure was measured weekly, so that there were six measurements during the 8 week period of angiotensin II or normal saline infusion.

### Morris Water Maze

On the 7th week of osmotic pump infusion of either normal saline or AngII, rats began behavioral testing. In a dimly lit room, a water tank (144 cm diameter) was filled with room temperature water, dyed with black non-toxic acrylic paint, and a target platform (12 cm diameter) was submerged below 3 cm of water. Rats were placed in a fixed start location and had to locate the hidden platform to be removed from the water tank (Figure 5A). The rats were given six 90 s learning trials (with 1 h inter-trial rest intervals) to learn the location of the submerged platform, aided by large distal visual cues; this learning schedule was adapted from Roof et al. (2001). Twenty-four hours after the last learning trial, the rats’ memory for the platform location was evaluated on a test trial. Rats were also evaluated on a 5-day series of delayed match-sample testing, to test for performance in more challenging spatial shifts (Figure 5B; Vorhees and Williams, 2006; Bizon et al., 2009). Each day, during the “sample trial,” a new start location and a new platform location was used. The rats are tested on these new spatial parameters 6 h later during the “match trial” and were assessed for improvement in their latency to find the platform. The 6 h delay was used to create a greater working memory challenge (Bizon et al., 2009). This was repeated with new start and platform locations each day over 5 days.

After MWM testing was complete, potentially confounding differences in visual perception or swim speed were evaluated on cued trials, wherein the location of the platform was visibly marked. All swim paths were tracked using ANYmaze tracking software, version 4.70 (Stoelting Company; Wood Dale, IL, United States), with a top-view webcam (C525, Logitech; Newark, CA, United States). The experimenter was not visible to the rats during testing.

### Open Field

The day after MWM testing was complete, rat exploratory behavior and anxiety was evaluated in the open field. Rats were placed in a square 45 cm open field with 40 cm black walls and a black floor and permitted to explore freely for 20 min. A top-view webcam was used for behavioral tracking with ANYmaze software, version 4.70 (Stoelting Company; Wood Dale, IL, United States). The experimenter was not visible to the rats during testing.

### Immunohistochemistry and Image Processing

Immediately after all behavioral testing was complete, before pump reservoirs were depleted, rats were euthanized, perfused with 200 ml of 0.01 PBS followed by 200 ml of 4% PFA, and brain tissue was collected and stored in 4% PFA for 24 h before transfer to 30% sucrose solution. 30 μm thick coronal sections were prepared from a subset of brains from each group (*n* = 8–10) using a cryostat (CryoStar NX50, Thermo Fischer Scientific; Ottawa, ON, Canada). DAB-mediated Immunohistochemistry (IHC) of free floating sections was performed with an ABC-HRP kit (Thermo Fischer Scientific #32020; Ottawa, ON, Canada), a 1:1,000 concentration of OX6 primary antibody for MHC-Class II to identify activated microglia (BD Biosciences #554926; Mississauga, ON, Canada) (Wong, 2013), a 1:2,000 concentration of GFAP primary antibody to identify reactive astrocytes (Sigma-Aldrich #G3893; Oakville, ON, Canada) (Sofroniew and Vinters, 2010), and a 1:500 concentration of 4G8 primary antibody for β-amyloid residues 18–23 (BioLegend, San Diego CA, United States) (Baghallab et al., 2018).

Stitched micrographs of slides were prepared using a 10× objective lens on an upright microscope (Nikon Eclipse Ni-E, Nikon DS Fi2 color camera, NIS Elements Imaging; Mississauga, ON, Canada). Anatomical regions of interest (cingulum, corpus callosum, internal capsule, and hippocampus) were captured at coronal sections: Bregma +2.00, +0.00, and -3.00 mm (Paxinos and Watson, 2007). Micrographs were processed and analyzed using ImageJ, version 1.50b; after regions of interest were outlined using the polygon tool, images were converted to 8-bit, processed using the subtract background command, and then thresholded with a fixed grayscale cutoff value of 237. Area coverage by DAB-positive cells (%) was recorded for each region of interest. The corpus callosum and cingulum were analyzed across three coronal planes and an average area coverage was calculated, weighted by cross-sectional area at each plane.

### Data Analysis

Two-Way ANOVA and linear regressions were used to evaluate the effects of genotype and AngII infusion using GraphPad Prism 7.0 software (La Jolla, CA). ANCOVA models were used to evaluate the effect of blood pressure as a continuous predictor variable with IBM SPSS version 23 (Armonk, NY, United States). The conservative Sidak’s *post hoc* analysis was used to compare outcome measures within genotype and infusate factors.

## Results

### Blood Pressure Elevation and Measurement

**FIGURE 1:**
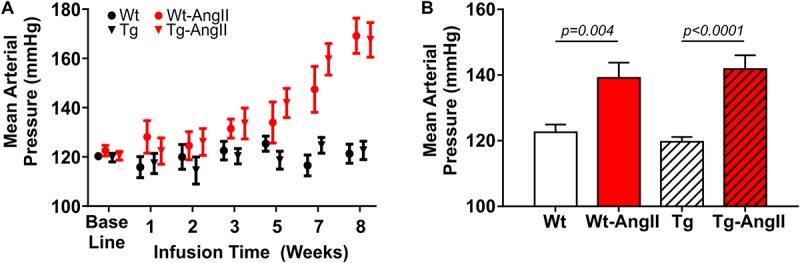
Mean arterial pressure during 8 weeks of normal saline or AngII infusion. **(A)** Baseline blood pressure and a progressively increasing mean arterial pressure over the course of AngII infusion is shown; blood pressure was not measured around the time of osmotic pump reimplantation (week 4). **(B)** Averaged over the 8-week infusion period, AngII infusion had a significant effect on mean arterial pressure. There were no differences in the genotypes’ response to AngII. *n* = 13–15; error bars indicate SEM.

Mean arterial pressure (MAP) increased over the 8 week AngII infusion period (Figure 1A). Averaged over the 8 week period, MAP was elevated by AngII infusion [Figure 1B; *p* < 0.0001, *F*(1,51) = 31.52; 2-Way ANOVA]. There were no genotype differences in response to AngII infusion and its effects on MAP, DBP, and SBP. *Post hoc* comparisons found a significant average MAP increase of 17 ± 5 mmHg and 22 ± 6 mmHg in Wt-AngII and Tg-AngII rats, respectively (±SE of difference; Wt-AngII: *p* = 0.004, *t* = 3.307, *df* = 51, Tg-AngII: *p* < 0.0001, *t* = 4.673, *df* = 51; Sidak’s test). DBP and SBP demonstrated the same pattern of significant changes; DBP respectively increased by 12 ± 5 mmHg and 21 ± 5 mmHg in Wt-AngII and Tg-AngII rats (Wt-AngII: *p* = 0.04, *t* = 2.446, *df* = 51; Tg-AngII: *p* < 0.0001, *t* = 4.547, *df* = 51), while SBP respectively increased by 19 ± 5 mmHg and 21 ± 5 mmHg Wt-AngII and Tg-AngII rats (Wt-AngII: *p* = 0.004, *t* = 4.039, *df* = 51; Tg-AngII: *p* < 0.0001, *t* = 4.604, *df* = 51; Sidak’s test). There were no group differences in baseline blood pressures, measured prior to pump implantation.

### Amyloid

**FIGURE 2:**
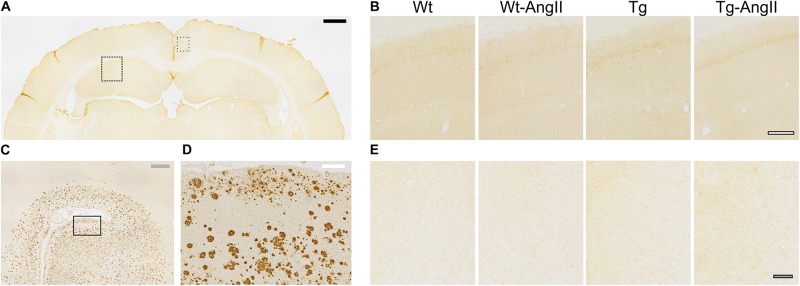
No hippocampal or cortical amyloid deposits observed in wildtype or transgenic rats. **(A)** Representative coronal cross section stained with DAB-mediated IHC with the 4G8 antibody for β-amyloid amino acid residues 17–24. Dashed rectangle indicates area of dorsal hippocampal tissue shown in **(B)** and dotted rectangle indicates area of cortical tissue shown in **(E)**; black bar = 1,000 μm. **(B)** Representative Wt, Wt-AngII, Tg, and Tg-AngII hippocampal tissue stained with 4G8 demonstrated no amyloid deposits; white bar outlined in black = 200 μm. **(C,D)** Representative human autopsy cortical tissue with clinical history of AD was used as a positive control for the 4G8 antibody. Rectangle in **(C)** indicates area of tissue shown in **(D)**; gray and white bars represent 1,000 and 200 μm, respectively. **(E)** Representative Wt, Wt-AngII, Tg, and Tg-AngII cortical tissue stained with 4G8 demonstrated no deposits of amyloid; gray bar in black outline = 100 μm.

Qualitative assessment revealed an absence of β-amyloid deposition in the hippocampal and cortical tissue of all experimental groups (Figure 2). This is in keeping with prior studies of naïve TgAPP21 rats and suggests that 8 weeks of blood pressure elevation by AngII infusion does not induce deposition of β-amyloid.

### Reactive Astrocytes

**FIGURE 3:**
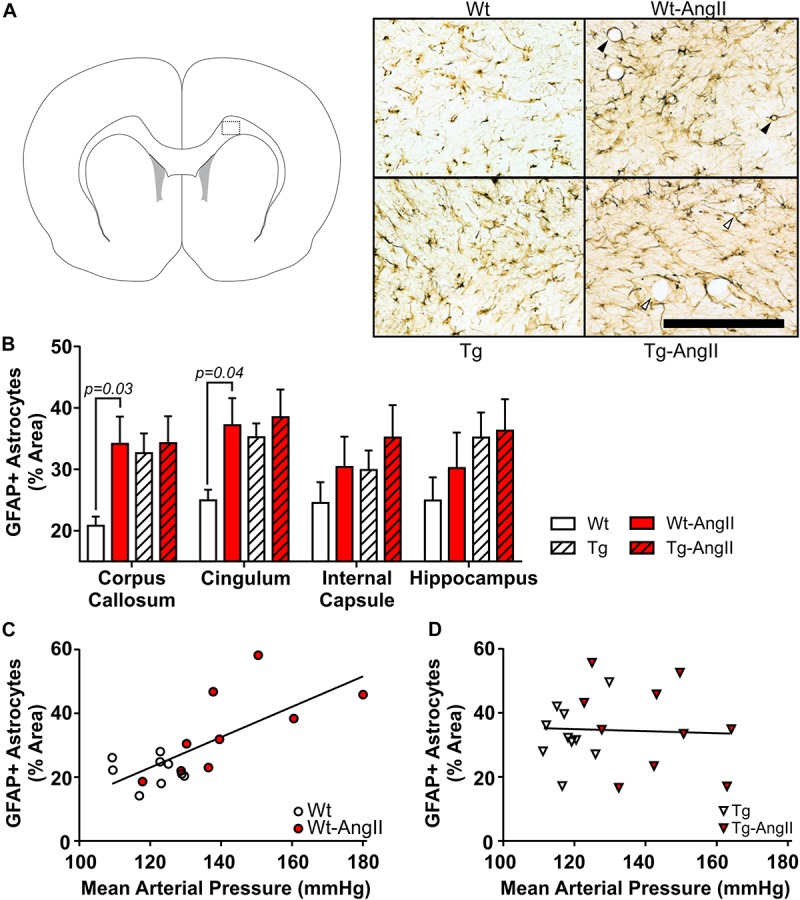
White matter astrocytosis increases linearly with blood pressure elevation in wildtype rats but is elevated in transgenic rats irrespective of blood pressure. Reactive astrocytes were identified using DAB-mediated IHC with a primary antibody for GFAP. **(A)** The schematic shows one of the coronal planes on which the corpus callosum was analyzed (Bregma + 2.0 mm). Representative images of 30 μm thick sections were taken from the dotted outline; black bar = 200 μm. Black triangles identify representative blood vessels associated with extensive astrocyte processes, as commonly observed in Wt-AngII rats; in comparison, white triangles identify representative blood vessels with relatively incomplete astrocyte engagement, as observed in Tg-AngII rats. **(B)** AngII infusion was found to have a significant effect on astrocyte activity in the corpus callosum and cingulum in Wt and Wt-AngII rats only. **(C)** Elevated mean arterial pressure linearly increased astrocytosis in the cingulum and corpus callosum of wildtype rats (*R*^2^ = 0.52, *p* = 0.0007), **(D)** but not transgenic rats. *n* = 9–10; error bars indicate SEM.

Reactive astrocytes were found throughout white matter, cortical, subcortical, and hippocampal regions. However, qualitative observations identified more variable degrees of astrocytosis in major white matter tracts (corpus callosum, cingulum, internal capsule) and the hippocampus, so these regions were selected for closer investigation (Figure 3). AngII infusion was found to have a significant effect on astrocyte activity in the corpus callosum and cingulum [Figure 3B; corpus callosum: *p* = 0.05, *F*(1,34) = 4.265; cingulum: *p* = 0.03, *F*(1,34) = 4.973; 2-Way ANOVA]. This was driven by significant differences between Wt and Wt-AngII rats (corpus callosum: *p* = 0.03, *t* = 2.537, *df* = 34; cingulum: *p* = 0.04, *t* = 2.426, *df* = 34; Sidak’s test). While area coverage by reactive astrocytes increased in the corpus callosum and cingulum of Wt-AngII rats, Tg rats also had elevated astrocytosis that did not increase further in Tg-AngII rats. Astrocytosis in the corpus callosum and cingulum was linearly correlated with MAP in wildtype rats (Figure 3C; *R*^2^ = 0.52, *p* = 0.0007, *df* = 18), but not in transgenic rats (Figure 3D). Regardless of whether the corpus callosum and cingulum were pooled or analyzed separately, similar significant relationships were identified and the slopes of the regressions between astrocytosis and MAP were significantly different between wildtype and transgenic rats. Moreover, genotype, MAP, and their interaction were found to have significant effects on astrocytosis in the corpus callosum and cingulum in an ANCOVA model with MAP as a continuous predictor [genotype: *p* = 0.009, *F*(1,34) = 7.568; MAP: *p* = 0.03, *F*(1,34) = 4.893; genotype × MAP: *p* = 0.02, *F*(1,34) = 6.407; ANCOVA). The same findings of significance were observed when the corpus callosum and cingulum were analyzed separately. Absolute MAP was found to be more informative than relative increases of MAP (as compared to baseline measurements prior to pump implantation), as relative change in MAP was not a significant predictor of astrocytosis. In Wt rats, blood pressure elevation increased white matter astrocyte reactivity to levels matching Tg and Tg-AngII rats. These findings suggest that white matter astrocyte reactivity was already saturated in Tg rats and could not increase further in response to elevated blood pressure.

In Wt-AngII rats, reactive astrocytes had extensive processes wrapping around blood vessels (Figure 3A). In contrast, Tg-AngII rats showed some increase of reactive astrocyte processes wrapping around blood vessels, but not as consistently as Wt-AngII. In comparison to Wt-AngII, astrocytic processes were qualitatively observed to be reduced in Tg-AngII around blood vessels ranging from 4 to 50 μm in diameter. This further supports the interpretation that astrocytes in Tg rats were already reactive at maximum capacity and could not respond to elevated blood pressure in Tg-AngII rats.

### Activated Microglia

**FIGURE 4:**
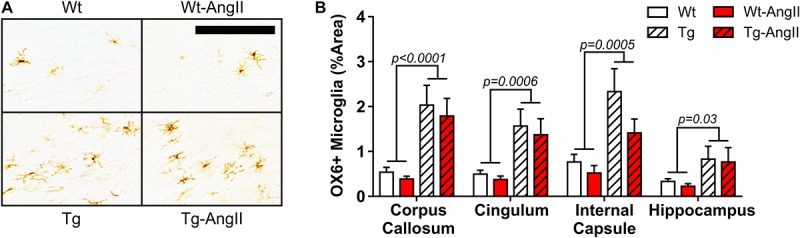
Increased white matter and hippocampal microglia activation in transgenic rats. **(A)** Representative images show activated pro-inflammatory microglia in the corpus callosum and cingulum, identified using DAB mediated IHC with the OX6 primary antibody for MHC Class II. 30 μm thick coronal sections; 200 μm bar. **(B)** Genotype was a significant factor in microglial activation in all regions analyzed. *n* = 8–10; error bars indicate SEM.

Activated microglia were found infrequently in Wt and Wt-AngII rats but they did appear consistently in major white matter tracts and the hippocampus of Tg and Tg-AngII rats (Figure 4A). Both Tg and Tg-AngII rats demonstrated a significant increase of microglia activation in the corpus callosum, cingulum, internal capsule, and hippocampus (Figure 4B; corpus callosum: *p* < 0.0001, *F*(1,33) = 22.02; cingulum: *p* = 0.006, *F*(1,33) = 14.26; internal capsule: *p* = 0.0005, *F*(1,33) = 15.33; hippocampus: *p* = 0.03, *F*(1,33) = 5.248). Neither AngII infusion nor MAP had any significant relationship with microglia activation in these regions.

### Open Field

**FIGURE 5:**
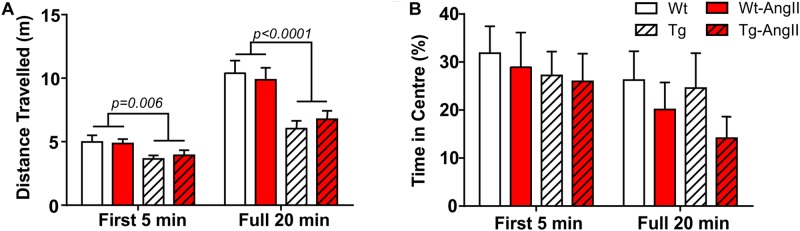
Reduced exploratory behavior in transgenic rats. **(A)** Tg and Tg-AngII traveled significantly less during both the first 5 min period of the test and during the full 20 min test, while AngII had no significant effect on distance traveled. **(B)** No significant group differences were observed in avoidance of the anxiogenic center of the open field. *n* = 13–15; error bars indicate SEM.

Exploratory behavior was evaluated by measuring the total distance traveled in the open field test; a subanalysis also evaluated the first 5 min period, which is typically more anxiogenic and also a period of greater exploration (Figure 5A). Genotype was a significant factor, with Tg and Tg-AngII rats having demonstrated less exploration [first 5 min: *p* = 0.006, *F*(1,51) = 8.342; full 20 min: *p* < 0.0001, *F*(1,51) = 22.39; 2-Way ANOVA]. AngII infusion did not have an effect on exploratory behavior. Time spent in the center of the field is a proxy measure of anxiety, where increased avoidance of the center is considered to reflect increased levels of anxiety. There were no significant group differences in this measure during either the first 5 min period or the full 20 min test duration (Figure 5B). Thus, anxiety is unlikely to have confounded behavioral measures.

### Morris Water Maze

**FIGURE 6:**
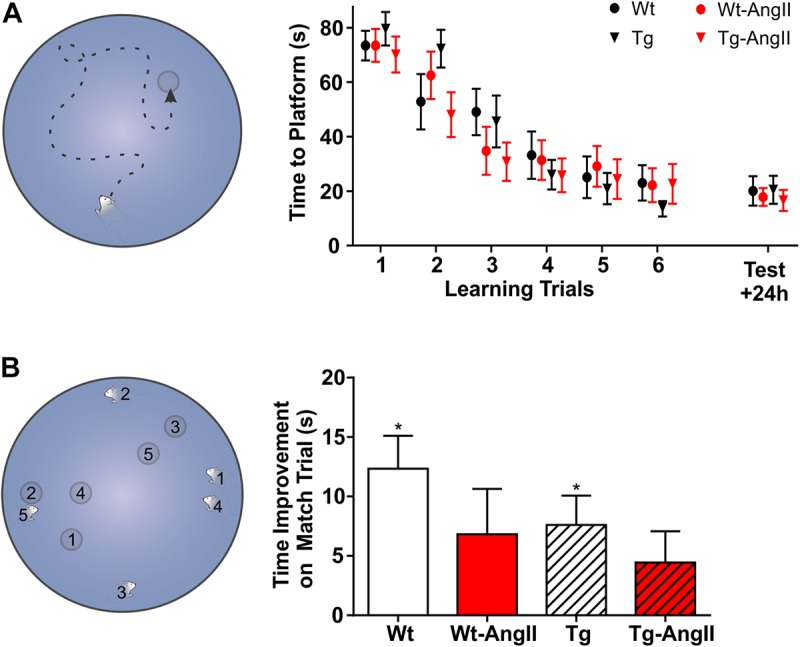
AngII infusion impaired executive functions. **(A)** With a fixed start location, rats were given six trials to learn the platform location; 24 h later, a test trial evaluated spatial memory. No significant group differences were observed on the learning or test trials. **(B)** Every day for 5 days, rats were challenged to learn a new platform location from a new start location. The corresponding number pairs on the MWM schematic indicate the start location and platform location on each day. After the first exposure to a new platform location on a “sample” trial, swim time improvement was measured on a 6 h delayed “match” trial. ^∗^Averaged across the five test days, Wt and Tg rats demonstrated a significant swim time improvement on the match trial (*p* < 0.03) but Wt-AngII and Tg-AngII rats did not, suggesting that AngII infusion impaired working memory and/or behavioral flexibility. *n* = 13–15; error bars indicate SEM.

No group differences were observed in spatial learning and memory (Figure 6A). However, AngII infusion appeared to impair performance on delayed match-sample testing (Figure 6B). Every day for 5 days, on the “sample” trial, rats began their swim from a new start location and had to find the new platform location. Swim time improvement was then evaluated on a 6 h delayed “match” trial. Averaged across the five test days, Wt and Tg rats demonstrated significant non-zero swim time improvements on the match trial (Wt: *p* = 0.003, *t* = 4.482, *df* = 12; Tg: *p* = 0.03, *t* = 3.082, *df* = 13; one-sample *t*-test with Bonferonni correction). In contrast, AngII infusion impaired rats of both genotypes, so that Wt-AngII and Tg-AngII did not demonstrate a significant swim time improvement. The frequently changing spatial parameters of delayed match-sample testing placed greater demands on spatial reference memory, working memory, and behavioral flexibility (Vorhees and Williams, 2006; Bizon et al., 2009). However, no group differences were observed in spatial reference memory (Figure 6A), so AngII infusion is more likely to have impaired working memory and/or behavioral flexibility. During cued trials, no differences were observed in swim time to platform nor swim speed, so visual acuity nor mobility were unlikely to have had any confounding effect.

## Discussion

Normotensive Tg rats had elevated baseline astrocyte reactivity in the corpus callosum and cingulum, while Tg-AngII rats did not show a further increase of astrocytosis. The increase of reactive astrocytes in Wt-AngII rats was accompanied with extensive astrocytic processes around blood vessels. The reactivity of astrocytes around blood vessels was less consistent and relatively incomplete in Tg-AngII. This suggested that astrocytes in AngII-infused transgenic rats did not develop a normal response to elevated blood pressure. Of note, these changes were observed in the absence of β-amyloid deposition. The role of transgenic hAPP in the insufficient response of TgAPP21 astrocytes is supported by previous findings of amyloid overwhelming astrocytes and inducing senescence in astrocytes (Bhat et al., 2012; Söllvander et al., 2016).

AngII infusion impaired both Wt-AngII and Tg-AngII rats in the MWM adaptation of a delayed match-sample test, a spatial task that tests working memory and behavioral flexibility, consistent with the clinical studies on the cognitive effects of hypertension (Raz et al., 2003; Vicario et al., 2005; Li et al., 2016). Previous studies have found histological and cognitive effects of AngII infusion or AngII blockade to be independent of blood pressure, which has been attributed to the central effects of AngII (Tedesco et al., 2002; Iadecola and Gorelick, 2004; Takeda et al., 2009; Capone et al., 2011; Hajjar et al., 2012; Takane et al., 2017), though these studies did not explicitly assess for white matter changes. It remains a possibility that high blood pressure alone, independently of AII, induces astrocytosis in the WM. In support of this interpretation, we found a linear relationship between MAP and astrocytosis in wildtype rats. Altogether, this suggests that there may be distinct benefits of both blood pressure management and central AngII blockade. Our findings contribute to this literature by identifying the prevention of white matter astrocytosis as a potentially important process and a therapeutic target in the management of high blood pressure or AngII blockade.

While AngII infusion did not affect activation of microglia in either genotype, Tg and Tg-AngII rats demonstrated significantly more pro-inflammatory activation of microglia in the corpus callosum, cingulum, internal capsule, and hippocampus, consistent with previous findings (Levit et al., 2019). This further indicates dysregulation in the cerebral tissue of TgAPP21 rats and implicates pro-inflammatory microglia as an important factor in the early pre-plaque stages of AD (Prokop et al., 2013; von Bernhardi et al., 2015). White matter microgliosis has also been identified as an important early factor of neurodegeneration in recent animal and human studies (Raj et al., 2017; Hase et al., 2018).

Future studies to identify specific molecular targets in the astrocyte, such as cytokines and other cell signaling factors, may prove to be crucial to managing the intersection of hypertension and AD-related pathology. As AngII has been shown to have histological and cognitive effects independent of blood pressure, further work with TgAPP21 rat model should consider non-hypertensive doses of AngII, alternate means of inducing blood pressure elevation, and AngII blockade to discern the role of AngII and blood pressure on white matter changes. Future preclinical research on the cerebral and cognitive effects of hypertension should also evaluate for structural white matter damage, including myelin quantification, capillary density, and blood-brain-barrier integrity. Functional white matter changes could be assessed through electrophysiological studies as well as the responsiveness of cerebral blood flow in white matter. Investigating anterograde and retrograde neuronal integrity and density in comorbid animal models would offer additional important insight into mixed AD and vascular dementia. More comprehensive assessment of processing speed as well executive function, including the subdomain of inhibition control, should also be pursued.

In the present study we demonstrate that in rat model of AD that expresses a pathogenic variant of hAPP, white matter perivascular astrocytes do not react to elevated blood pressure in a normal fashion. This captures an important interaction that may present in the comorbid burden of hypertension and AD. The comorbidity of hypertension and pathogenic hAPP expression may have an overwhelming effect on astrocytes, which are central to the maintenance of the neurovascular unit. Regardless of genotype, we found that AngII-infused impaired the working memory and behavioral flexibility of rats with elevated blood pressure; further work will be needed to discern whether this effect was mediated by blood pressure elevation or by the central effects of AngII. Regardless of genotype, we found that AngII-infused impaired the working memory and behavioral flexibility of rats with elevated blood pressure; further work will be needed to discern whether this effect was mediated by blood pressure elevation or by the central effects of AngII. This will inform the ongoing study of the role of hypertension in white matter disease and executive dysfunction. Characterizing the potential dysregulations induced by hypertension and pathogenic hAPP will be crucial to refining ongoing research on the neuroprotective effects of antihypertensive treatment.

## Data Availability Statement

The datasets generated for this study are available on request to the corresponding author.

## Ethics Statement

Animal ethics and procedures were approved by the Animal Care Committee at Western University (protocol 2014-016).

## Author Contributions

AL, VH, and SW made substantial contributions to the conception and design of the work. AL, SC, OH, QL, and SW made substantial contributions in the acquisition and analysis of the data. AL, YA, CA, and SW drafted the manuscript. All authors contributed to manuscript revision, read, and approved the submitted version.

## Conflict of Interest

The authors declare that the research was conducted in the absence of any commercial or financial relationships that could be construed as a potential conflict of interest.
